# Performance of surveillance case definitions for respiratory syncytial virus infections through the sentinel influenza surveillance system, Portugal, 2010 to 2018

**DOI:** 10.2807/1560-7917.ES.2019.24.45.1900140

**Published:** 2019-11-07

**Authors:** Emma Sáez-López, Pedro Pechirra, Inês Costa, Paula Cristóvão, Patrícia Conde, Ausenda Machado, Ana Paula Rodrigues, Raquel Guiomar

**Affiliations:** 1 Department of Infectious Diseases, National Health Institute Doutor Ricardo Jorge, Lisbon, Portugal; 2 European Public Health Microbiology Training (EUPHEM), European Centre for Disease Prevention and Control (ECDC), Stockholm, Sweden; 3 Department of Epidemiology, National Health Institute Doutor Ricardo Jorge, Lisbon, Portugal

**Keywords:** respiratory syncytial virus, clinical predictors, case definition, sentinel influenza surveillance system, Portugal

## Abstract

**Background:**

Well-established influenza surveillance systems (ISS) can be used for respiratory syncytial virus (RSV) surveillance. In Portugal, RSV cases are detected through the ISS using the European Union (EU) influenza-like illness (ILI) case definition.

**Aim:**

To investigate clinical predictors for RSV infection and how three case definitions (EU ILI, a modified EU acute respiratory infection, and one respiratory symptom) performed in detecting RSV infections in Portugal.

**Methods:**

This observational retrospective study used epidemiological and laboratory surveillance data (October 2010–May 2018). Associations between clinical characteristics and RSV detection were analysed using logistic regression. Accuracy of case definitions was assessed through sensitivity, specificity, and area under the receiver operating characteristic curve (AUC). A 0.05 significance level was accepted.

**Results:**

The study involved 6,523 persons, including 190 (2.9%) RSV cases. Among 183 cases with age information, RSV infection was significantly more frequent among individuals < 5 years (n = 23; 12.6%) and ≥ 65 years (n = 45; 24.6%) compared with other age groups (p < 0.0001). Cough (odds ratio (OR): 2.4; 95% confidence interval (CI): 1.2–6.5) was the best RSV-infection predictor considering all age groups, while shortness of breath was particularly associated with RSV-positivity among ≤ 14 year olds (OR: 6.7; 95% CI: 2.6–17.4 for 0–4 year olds and OR: 6.7; 95% CI: 1.5–28.8 for 5–14 year olds). Systemic symptoms were significantly associated with RSV-negative and influenza-positive cases. None of the case definitions were suitable to detect RSV infections (AUC = 0.51).

**Conclusion:**

To avoid underestimating the RSV disease burden, RSV surveillance within the Portuguese sentinel ISS would require a more sensitive case definition than ILI and, even a different case definition according to age.

## Introduction

The human respiratory syncytial virus (RSV) is a major cause of morbidity and mortality worldwide since it is the predominant viral agent affecting the respiratory tract, causing acute, sometimes fatal lower respiratory tract infections in infants, young children and the elderly [1]. In fact, RSV has been associated with a substantial disease burden in adults, especially in the elderly, with an estimation of 1.5 million episodes of acute respiratory infection (ARI) in industrialised countries in 2015 [2-4]. Moreover, according to a study in the same year, the global burden of RSV-associated acute lower respiratory infection has been estimated at 33.1 million annually resulting in over 3.2 million severe illness that required hospitalisation in children younger than 5 years [5].

Currently, the available options for clinical management of RSV disease are symptomatic supportive care [6] as well as Palivizumab. Palivizumab is a humanised antibody against the F glycoprotein of the virus. It prevents RSV infection and has been shown to reduce the number of hospitalised cases by half [7]. It can also be employed to treat RSV but it does not reduce RSV mortality and its use is limited to selected populations in high-resource settings [6,8-10]. Several RSV vaccines are progressing in phase III clinical trials and RSV vaccines are expected to become available in the coming 5 to 10 years. With this perspective, evidence-based support for vaccination policies at the national, regional and global levels is necessary and, in 2015, the World Health Organization (WHO) made it a high priority to establish robust age-specific estimates of those affected by RSV and globally-compatible RSV-disease-burden surveillance systems [11]. Many countries however detect RSV infections within existing surveillance systems for influenza [12-13]. Moreover, one of the challenges to implement a global RSV surveillance system is the lack of a uniform case definition. Influenza case definitions may be less sensitive for RSV and, consequently, have the potential to underestimate the RSV burden [14]. Indeed, a broader ARI case definition, which includes a sudden onset of symptoms and at least one respiratory symptom (cough, sore throat, shortness of breath or coryza), has been considered to be more suitable for capturing RSV infections [1,15].

In Portugal, RSV cases are detected using the standard European Union (EU) influenza-like illness (ILI) case definition through the influenza surveillance system (ISS). The EU ILI case definition includes sudden onset of symptoms, at least one respiratory symptom (cough, sore throat, shortness of breath), and at least one systemic symptom (fever or feverishness, malaise, headache or myalgia) [15]. Therefore, we aimed to evaluate signs and symptoms as clinical predictors of RSV, and to estimate the sensitivity and specificity of three case definitions, including EU ILI, a modified EU ARI [15], and one respiratory symptom, for detecting RSV infections through the country’s sentinel ISS.

## Methods

### Portuguese Influenza Surveillance System

The Portuguese ISS comprises a sentinel and a non-sentinel component. The sentinel component, which is the sentinel ISS, exists since 1990 and is composed by the General Practitioners’ (GP) Sentinel Network, GP from the I-MOVE’s Euro EVA project (which is a project to monitor the effectiveness of influenza vaccine in the EU at a community-level) and the Emergency and Obstetric Departments Networks. The sentinel ISS includes primary healthcare centres, general hospitals with paediatric and adult emergency rooms and medical wards, and one reference paediatric hospital in Lisbon. Age and sex distribution of the population under observation in the GP Sentinel Network is similar to that of the Portuguese population [16]. Using a data reporting form, the sentinel ISS weekly reports demographic, clinical and laboratory data of individuals tested for influenza and other respiratory viruses to the National Reference Laboratory for Influenza and other Respiratory Viruses at the National Institute of Health Doutor Ricardo Jorge (INSA) during the influenza season. The influenza season comprises the period between week 40 (October) and week 20 (May) of the next year. In addition, the sentinel ISS sends nasopharyngeal swabs for influenza and other respiratory viruses diagnosis to INSA.

The non-sentinel component of the ISS is formed by the Portuguese Laboratory Network for the Diagnosis of Influenza Infection (PLNDII), which comprises 14 hospital-based laboratories and is coordinated by INSA. The PLNDII was established in 2009 in response to the influenza pandemic to monitor trends in influenza and other respiratory viruses, including adenovirus, enterovirus D68, human coronavirus, human metapneumovirus, parainfluenza (1, 2 and 3), rhinovirus, and RSV A and B. It reports to INSA the same data as the sentinel ISS with the exception of clinical characteristics.

### Study population and data

Individuals tested through the sentinel ISS from 2010 to 2018 in Portugal for any respiratory virus (adenovirus, enterovirus D68, human coronavirus, human metapneumovirus, parainfluenza (1, 2 and 3), rhinovirus and RSV A and B) with epidemiological, clinical and laboratory data were included in the study. Data were collected based on the detection of influenza cases through the sentinel ISS using the EU ILI case definition. Clinical characteristics were interpreted by the doctors or reported by the patients (or their parents if the children could not). Missing characteristics were indicated as unknown or not reported. Persons were excluded from the study if they had the following missing clinical characteristics: sudden onset of symptoms or ≥ 2 respiratory symptoms or ≥ 2 systemic symptoms. RSV detection was performed using real-time PCR as previously described by Gunson et al. [17], with a slight modification of cycling conditions (30 min at 50 °C, 2 min at 95 °C, followed by 45 cycles at 95 °C for 8 s and 60 °C for 1 min). 

### Ethical statement

Data used within this study were anonymised and were collected: (i) in the scope of epidemiological surveillance for which submission to an ethical committee is not required and (ii) specific projects including GP Sentinel Network and I-MOVE’s Euro EVA, which had already been approved by the Health Ethic Committee of INSA.

### Case definitions tested

The EU ILI case definition is used in the sentinel ISS. However, not all individuals fulfilled its criteria. Therefore, in order to validate the accuracy of RSV detection through the ISS, we decided to test three different case definitions (Table 1): (i) EU ILI, (ii) a modified EU case definition for ARI (ARI-like), and (iii) only one respiratory symptom of the three included in ARI-like/ILI case definitions. ARI-like and only one respiratory symptom case definitions were created based on the clinical characteristics that were reported by the sentinel ISS.

**Table 1:** Case definitions tested for accuracy of respiratory syncytial virus detection through the sentinel influenza surveillance system, Portugal, 2010–2018

Case definition	Sudden onset of symptoms	Respiratory symptoms	Systemic symptoms
ILI (EU, 2012)	Yes	At least one among: cough, sore throat or shortness of breath	At least one among: fever or feverishness, malaise, headache, myalgia
ARI-like^a^ (EU, 2012)	Yes	At least one among: cough, sore throat or shortness of breath	No
One respiratory symptom	No	At least one among: cough, sore throat or shortness of breath	No

ARI: acute respiratory infection; EU: European Union; ILI: influenza-like illness.

**^a^** The EU case definition for ARI includes coryza in the group of respiratory symptoms, as well as the clinician’s judgement that the illness is due to an infection. In this study, coryza and the clinician’s judgement were not included in the ARI-like case definition because they were not collected in the Portuguese Influenza Surveillance System.

### Data analysis

Retrospective analyses were performed on data from individuals included in the study. Demographic characteristics were described using percentages. Associations were assessed using the chi-squared test. For the sudden onset of symptoms, each symptom and the three case definitions, we calculated the odds ratios (OR) and 95% confidence intervals (CI) to evaluate their association with laboratory-confirmed RSV by bivariate logistic regression. Moreover, we calculated the relative risk ratio (RRR) and 95% CI for laboratory-confirmed RSV and influenza using multinomial analysis for three groups: (i) RSV-positive cases, (ii) influenza-positive cases and (iii) a reference group that included negative cases for any respiratory virus. Case definition performance characteristics, including sensitivity, specificity, and area under the receiver operator characteristic curve (AUC), were evaluated according to the age group [18]. Tests with p value < 0.05 were considered statistically significant. The data analysis was performed using STATA 12.

## Results

### Selection of study patients and demographic characteristics

During the influenza seasons between 2010 and 2018, 7,085 individuals were tested for any respiratory viral agent (Figure). Of these, 562 were excluded from this study due to missing clinical characteristics. Remarkably, myalgia and malaise were missing in children aged ≤ 14 years. Data on age were not recorded for 97 individuals. For the 6,426 persons with known age, the majority were 15–64 years old (73.0%; n = 4,689), followed by ≥ 65 years old (14.9%; n = 961), 5–14 years old (9.3%; n = 596), and < 5 years old (2.8%; n = 180). The female–male ratio was 1.36:1. Among the 6,523 patients included in the study, 66.3% (n = 4,322) were laboratory-confirmed for any respiratory virus and a significant (p < 0.0001) difference was found according to the age group. Observed frequencies were higher than those ones expected if respiratory virus infection and age were independent among children aged < 5 years (154.0 vs 118.9) and 5–14 years (444.0 vs 393.8) (data not shown). Moreover, 5,142 (78.9%) and 5,162 (79.1%) fulfilled the ILI and ARI-like case definitions, respectively.

**Figure:**
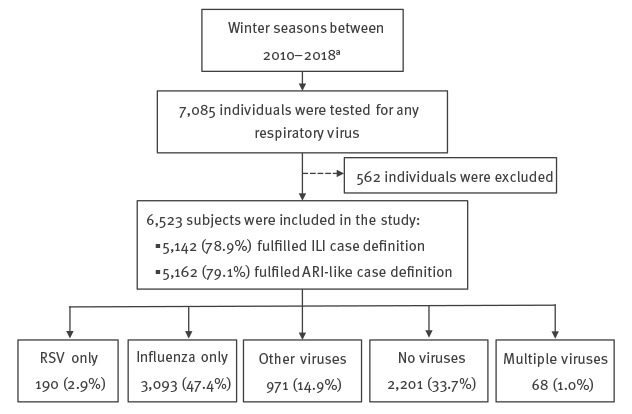
Respiratory virus surveillance data obtained through the sentinel influenza surveillance system, Portugal, 2010–2018 (n = 7,085 individuals)

ARI: acute respiratory infection; ILI: influenza-like illness; RSV: respiratory syncytial virus.

^a^ The sampling method differed depending on the season, but no significant difference in RSV prevalence in function of the season was found.

Among RSV-positive cases the female–male ratio seemed to be higher (1.85:1) than among negative counterparts (1.37:1) (Table 2). Most of RSV-positive cases (58.5%; 107/183) were adults between 15 and 64 years old. However, observed frequencies of RSV infection were statistically significantly higher (p < 0.0001) in children aged < 5 years (12.6%; 23/183) and adults ≥ 65 years (24.6%; 45/183) in comparison with other age groups.

**Table 2:** Demographic characteristics of individuals detected positive and negative for respiratory syncytial virus, who were tested through the sentinel influenza surveillance system, Portugal, 2010–2018 (n = 6,455 persons)^a^

Demographic characteristics^b^	Virus infection^a^			
	RSV-positive cases		RSV-negative cases	
	n	%	n	%
**Age group in years**				
0–4^c^ (n = 171)	23	12.6	148	2.4
5–14 (n = 585)	8	4.4	577	9.3
15–64 (n = 4,652)	107	58.5	4,545	73.6
≥ 65^c^ (n = 951)	45	24.6	906	14.7
**Total (n = 6,359)**	**183**	**100.0**	**6,176**	**100.0**
**Sex**				
Male (n = 2,698)	66	35.1	2,632	42.1
Female (n = 3,736)	122	64.9	3,614	57.9
**Total (n = 6,434)**	**188**	**100.0**	**6,246**	**100.0**

RSV: Respiratory syncytial virus.

^a^ Of 6,523 individuals included in the study, those with mixed infections (n = 68) were excluded in this Table.

^b^ Cases with missing age or sex were excluded from the analyses by age or sex respectively. Age was missing for seven RSV-positive and 89 RSV-negative cases; sex was missing for two RSV-positive and 19 RSV-negative cases.

^c^ Observed frequencies were higher than expected among these age groups: 0–4 and ≥ 65 years (p < 0.0001).

### Clinical predictors of respiratory syncytial virus infection

The most predictive clinical symptoms for laboratory-confirmed RSV were cough (OR: 2.4; 95% CI: 1.2–6.5) and shortness of breath (OR: 2.1; 95% CI: 1.6–2.9), whereas fever or feverishness (OR: 0.4; 95% CI: 0.3–0.6), headache (OR: 0.7; 95% CI: 0.5–0.9) and myalgia (OR: 0.4; 95% CI: 0.3–0.5) were negatively associated with RVS-positive cases (Table 3). When we stratified by age groups, the clinical predictors for RSV-positive cases differed according to the age group. Adults ≥ 65 years old did not show any significant clinical predictor. Shortness of breath was more likely in RSV-positive than in RSV-negative cases aged ≤ 14 years (OR: 6.7 for 0–4 year olds; OR: 6.7 for 5–14 year olds). Finally, the ILI (OR: 2.5; 95% CI: 1.3–4.9) and ARI-like (OR: 2.5; 95% CI: 1.3–4.8) case definitions were only significantly associated with RSV-positive cases in 15–64 year olds.

**Table 3:** Clinical characteristics associated with laboratory-confirmed respiratory syncytial virus cases detected through the sentinel influenza surveillance system using bivariate logistic regression, Portugal, 2010–2018 (n = 190 cases)

Clinical characteristics	Total (n = 190)				Age group (years)^a^															
					0–4 (n = 23)				5–14 (n = 8)				15–64 (n = 107)				≥ 65 (n = 45)			
	n	%	Crude OR	95% CI	n	%	Crude OR	95% CI	n	%	Crude OR	95% CI	n	%	Crude OR	95% CI	n	%	Crude OR	95% CI
Sudden onset of symptoms	157	82.6	1.1	0.7–1.5	13	56.5	0.4	0.16–1.00^b^	8	100.0	1.0	NA	97	90.7	2.1	1.1–4.0^b^	34	75.6	0.8	0.4–1.7
**Symptoms**																				
Fever or feverishness	129	67.9	0.4	0.3–0.6^b^	22	95.7	1.1	0.1–9.3	8	100.0	1.0	NA	70	65.4	0.3	0.2–0.5^b^	24	53.3	0.6	0.3–1.2
Malaise	164	86.3	0.7	0.44–1.0	17	73.9	1.0	0.4–2.8	7	87.5	1.4	0.2–11.3	97	90.7	0.9	0.4–1.8	39	86.7	0.7	0.3–1.6
Headache	123	64.7	0.7	0.5–0.9^b^	3	13.0	0.3	0.1–1.0	3	37.5	0.2	0.0–1^b^	84	78.5	1.1	0.7–1.7	28	62.2	1.0	0.5–1.8
Myalgia	138	72.6	0.4	0.3–0.5^b^	7	30.4	0.5	0.2–1.2	4	50.0	0.3	0.1–1.3	88	82.2	0.4	0.2–0.6^b^	36	80.0	0.9	0.4–1.9
Cough	180	94.7	2.4	1.2–6.5^b^	23	100.0	1.0	NA	7	87.5	0.9	0.1–7.3	99	92.5	1.5	0.7–3.2	44	97.8	1.0	NA
Sore throat	143	75.3	1.2	0.9–1.7	15	65.2	0.6	0.2–1.5	2	25.0	0.1	0.0–0.4^b^	90	84.1	2.3	1.3–4.2^b^	32	71.1	1.3	0.6–2.5
Shortness of breath	74	38.9	2.1	1.6–2.9^b^	12	52.2	6.7	2.6–17.4^b^	3	37.5	6.7	1.5–28.8^b^	31	29.0	1.5	1.0–2.3	25	55.6	1.7	0.9–3.2
**Case definition**																				
EU ILI	154	81.1	1.2	0.8–1.7	13	56.5	0.5	0.2–1.2	7	87.5	1.7	0.2–13.7	97	90.7	2.5	1.3–4.9^b^	34	75.6	1.0	0.5–1.9
ARI-like	156	82.1	1.2	0.8–1.8	13	56.5	0.5	0.2–1.1	7	87.5	1.6	0.2–13.5	97	90.7	2.5	1.3–4.8^b^	34	75.6	0.9	0.5–1.9
One respiratory symptom	189	99.5	6.1	0.9–43.8	23	100.0	1.0	NA	7	87.5	0.2	0.3–2.0	107	100.0	1.0	NA	45	100.0	1	NA

ARI: acute respiratory infection; CI: confidence interval; EU: European Union; ILI: influenza-like illness; NA: not applicable; OR: odds ratio.

^a^ Age was not known for seven of the 190 patients infected with respiratory syncytial virus.

^b^ p value < 0.05.

Multinomial analysis showed that cough (RRR: 4.1; 95% CI: 2.1–8.1) and shortness of breath (RRR: 1.8; 95% CI: 1.3–2.5) were significantly associated (p < 0.0001) with RSV-positive cases whereas fever or feverishness (RRR: 0.6; 95% CI: 0.4–0.8) and myalgia (RRR: 0.5; 95% CI: 0.3–0.7) were negatively associated (Table 4). With the exception of malaise and sore throat, which were not significant, and shortness of breath (RRR: 0.7; 95% CI: 0.6–0.8), all clinical characteristics, including the EU ILI, the ARI-like and the one respiratory symptom case definitions, were significantly associated with influenza-positive cases.

**Table 4:** Comparison of clinical characteristics of respiratory syncytial virus-positive and influenza-positive groups with a group negative for any respiratory virus using multinomial analysis, Portugal, 2010–2018 (n = 5,484 persons)

Clinical characteristics	Viral respiratory infection^a^					
	RSV			Influenza		
	RRR	95% CI	p value	RRR	95% CI	p value
Sudden onset of symptoms	1.2	0.8–1.8	0.361	1.2	1.08–1.43	0.002
**Symptoms**						
Fever or feverishness	0.6	0.4–0.8	0.002	3.3	2.7–4.1	< 0.0001
Malaise	0.7	0.4–1.19	0.114	1.2	1.0–1.5	0.036
Headache	0.8	0.6–1.1	0.265	1.6	1.4–1.8	< 0.0001
Myalgia	0.5	0.3–0.7	< 0.0001	1.7	1.4–2.0	< 0.0001
Cough	4.1	2.1–8.1	< 0.0001	3.6	3–4.4	< 0.0001
Sore throat	1.3	0.9–1.9	0.112	1.1	1–1.3	0.108
Shortness of breath	1.8	1.3–2.5	< 0.0001	0.7	0.6–0.8	< 0.0001
**Case definition**						
ILI	1.4	1.0–2.1	0.063	1.5	1.3–1.7	< 0.0001
ARI-like	1.5	1.0–2.2	0.044	1.4	1.3–1.6	< 0.0001
One respiratory symptom	10.1	1.4–73	0.022	2.8	2.0–3.9	< 0.0001

ARI: acute respiratory infection; CI: confidence interval; ILI: influenza-like illness; RRR: relative risk ratio; RSV: respiratory syncytial virus.

^a^ Comparison with the reference group, which comprises individuals testing negative for any respiratory virus.

All persons were detected through the Portuguese sentinel Influenza Surveillance System.

### Performance of the influenza-like illness, acute respiratory infection-like and one respiratory symptom case definitions

When testing the performance of the case definitions, one respiratory symptom had the highest sensitivity for detection of RSV with 99.5%, whereas the ILI and ARI-like case definitions revealed the highest specificities with 21.1% and 20.8%, respectively (Table 5). AUC was 0.51 in all case definitions.

**Table 5:** Sensitivity, specificity and area under the curve of the tested case definitions for detection of respiratory syncytial virus through the sentinel influenza surveillance system, by age, Portugal, 2010–2018 (n =6,455 persons)

Age in years	EU ILI % (95% CI)	ARI-like % (95% CI)	One respiratory symptom % (95% CI)
**Sensitivity**			
0–4	56.5 (34.5–76.8)	56.5 (34.5–76.8)	100 (85.2–100)
5–14	87.5 (47.3–99.7)	87.5 (47.3–99.7)	87.5 (47.3–99.7)
15–64	90.7 (83.5–95.4)	90.7 (83.5–95.4)	100 (96.6–100)
≥ 65	75.6 (60.5–87.1)	75.6 (60.5–87.1)	100 (92.1–100)
**Total**	**81.1 (74.7–86.4)**	**82.1 (75.9–87.3)**	**99.5 (97.1–100)**
**Specificity**			
0–4	27.7 (20.7–35.7)	26.4 (19.5–34.2)	2.7 (0.7–6.8)
5–14	19.2 (16.1–22.7)	19.1 (15.9–22.5)	3.3 (2.8–3.8)
15–64	20.7 (19.5–21.9)	20.5 (19.4–21.7)	3.3 (2.8–3.8)
≥ 65	23.8 (21.1–26.8)	23.1 (20.4–26)	2.5 (1.6–3.8)
**Total**	**21.1 (20.1–22.1)**	**20.8 (19.8–21.9)**	**3.1 (2.7–3.6)**
**AUC**			
0–4	0.42 (0.31–0.53)	0.41 (0.30–0.52)	0.51 (0.50–0.53)
5–14	0.53 (0.41–0.66)	0.53 (0.41–0.66)	0.45 (0.33–0.58)
15–64	0.56 (0.53–0.59)	0.56 (0.53–0.58)	0.52 (0.51–0.52)
≥ 65	0.50 (0.43–0.56)	0.49 (0.43–0.56)	0.51 (0.51–0.52)
**Total**	**0.51 (0.48–0.54)**	**0.51 (0.49–0.54)**	**0.51 (0.51–0.52)**

ARI: acute respiratory infection; AUC: area under curve; CI: confidence interval; EU: European Union; ILI: influenza-like illness.

Concerning the analysis by age group, for children < 5 years old the ILI and ARI-like case definitions presented the lowest sensitivity (56.5%) and the highest specificity (27.7% and 26.4%, respectively). Only one respiratory symptom as case definition showed the highest sensitivity (100%) in all age groups, with the exception of the 5–14 years’ age group. This definition had the lowest specificity with a range from 2.5% to 3.3% depending on the age group. The ILI and ARI-like case definitions presented the highest AUC in the 15–64 years’ age group (0.56 for both definitions) and the lowest in the children < 5 years old (0.42 for ILI and 0.41 for ARI-like).

## Discussion

Although many studies regarding RSV have focused on children aged under 5 years and elderly adults, little is known regarding RSV among patients of all ages. This study evaluated the clinical characteristics and the performance of three different case definitions for the diagnosis of RSV infection based on epidemiological and laboratory data from 2010 to 2018 in Portugal.

In comparison with other age groups, laboratory-confirmed respiratory infection frequency in children aged ≤ 14 years was significantly higher than what would be expected if respiratory virus infection and age were independent. Moreover, RSV infection was significantly associated with two age groups, 0–5 years and ≥ 65 years, which is consistent with RSV infection being considered as one of the most common causes of respiratory tract infection during childhood as well as among elderly adults or adults with underlying medical conditions [19].

The clinical characteristics associated with RSV-positive cases varied across age groups, which suggested that a different case definition according to age might be suitable. Among respiratory symptoms, shortness of breath was significantly associated with being RSV-positive among children aged ≤ 14 years, whereas cough was most common (94.7%) and significantly associated with RSV infection overall. Nonetheless, no significant difference in the frequency of cough, either positive or negative, was found between age groups when we stratified by age, which differed from previous studies that found cough associated with RSV infection in children under 5 years old [12,20-22]. Among the systemic symptoms, fever has always been controversial as to its inclusion in the case definition for RSV infection [23]. In our study, fever or feverishness was very frequently observed among RSV-positive cases ≤ 14 years old (95.7–100%). However, no significant difference between this age group and others, either positive or negative, was found, which was in agreement with Nyawanda et al. [12] but not with other studies [14,20,21]. Moreover, among the 15–64 and ≥ 65 year olds, the observed frequencies (53.3–65.4%) of this symptom were similar to previous studies conducted in adults [24,25] and negatively associated with RSV-positive cases.

Overall in the current study, good clinical predictors for laboratory-confirmed RSV infection were respiratory symptoms including cough and shortness of breath whereas systemic symptoms were negatively associated. These findings were also in agreement with the results from the multinomial analysis. In this analysis, taking respectively as a reference persons testing negative for any respiratory virus, several considerable differences were found in the presentation of influenza and RSV, with all clinical characteristics considered in the EU ILI case definition significantly associated with influenza-positive cases with the exception of malaise, sore throat and shortness of breath. Therefore, it may be easier to diagnose influenza than RSV infection based on the considered clinical characteristics and case definitions in this study.

Much of what is known about RSV globally comes from surveillance systems that use case definitions intended for influenza, which are not optimised for RSV detection. In our study, we demonstrated that the EU ILI case definition, which is the one used in the current Portuguese sentinel ISS, was not accurate for RSV detection, and in fact, it was only a good clinical predictor in the 15–64 years’ age group. Furthermore, none of the evaluated three case definitions was able to discriminate among RSV-positive and negative cases with sufficiently high sensitivity and specificity to be clinically reliable because all these definitions presented an AUC of 0.51. However, the optimal case definition should be determined considering the epidemiological context, available resources, study population and the objective for which it is being used. Only one respiratory symptom showed the highest sensitivity with 99.5%, which may be very good to better understand burden, seasonality and mortality associated with RSV infection. In fact, a very high proportion of hospitalisations are due to respiratory symptoms [21]. For vaccine effectiveness studies, a highly specific case definition would be more suitable [26,27] and the ILI and ARI-like case definitions showed higher specificities (21.1% and 20.8%, respectively) than only one respiratory symptom, albeit they were low in comparison with previous studies in hospitalised children [12].

In interpreting our results, the following limitations should be taken into consideration. Firstly, missing symptoms of myalgia and malaise were mostly among individuals aged under 14 years. Secondly, the number of persons of this age group was very low in comparison with other age groups, with only 21 children under 1 year old included in the study, thus not allowing further age-stratified analyses in the 0−14 year-old age group. Thirdly, the analysis did not differentiate between inpatients and outpatients and, indeed, most collected information was from outpatients, whose underlying risk factors were unknown. Finally, data were collected based on EU ILI case definition and thereby, individuals presenting only one respiratory symptom might have not been selected to test for any respiratory virus resulting in a selection bias. Therefore, these findings may not be representative for inpatient respiratory disease surveillance and the most severe spectrum of cases with RSV infection. However, we believe that this study may contribute to decide the best option for RSV surveillance in Portugal, especially at a community level, and may help support public health strategies and interventions at a European and global level regarding the prophylaxis and treatment options.

In Portugal, the most feasible option would be to include RSV surveillance within the existing sentinel ISS. We demonstrated that the current EU ILI case definition was not suitable to detect RSV infections and indeed, a more sensitive case definition to avoid the underestimation of RSV burden disease and a different case definition according to age would be more appropriate. Moreover, hospital-based surveillance, especially with a focus on paediatric patients, including paediatric intensive care units, should be specifically enhanced for RSV.

## References

[1] World Health Organization (WHO). WHO strategy to pilot global respiratory syncytial virus surveillance based on the global influenza surveillance and response system (GISRS). Geneva: WHO; 2017. Available from: https://apps.who.int/iris/bitstream/handle/10665/259853/9789241513203-eng.pdf?sequence=1&isAllowed=y

[2] Shi T Denouel A Tietjen AK Campbell I Moran E Li X RESCEU Investigators Global Disease Burden Estimates of Respiratory Syncytial Virus-Associated Acute Respiratory Infection in Older Adults in 2015: A Systematic Review and Meta-Analysis. J Infect Dis. 2019;jiz059. 10.1093/infdis/jiz059 30880339

[3] Falsey AR McElhaney JE Beran J van Essen GA Duval X Esen M Respiratory syncytial virus and other respiratory viral infections in older adults with moderate to severe influenza-like illness. J Infect Dis. 2014;209(12):1873-81. 10.1093/infdis/jit839 24482398 PMC4038137

[4] Fleming DM Taylor RJ Lustig RL Schuck-Paim C Haguinet F Webb DJ Modelling estimates of the burden of Respiratory Syncytial virus infection in adults and the elderly in the United Kingdom. BMC Infect Dis. 2015;15(1):443. 10.1186/s12879-015-1218-z 26497750 PMC4618996

[5] Shi T McAllister DA O’Brien KL Simoes EAF Madhi SA Gessner BD RSV Global Epidemiology Network Global, regional, and national disease burden estimates of acute lower respiratory infections due to respiratory syncytial virus in young children in 2015: a systematic review and modelling study. Lancet. 2017;390(10098):946-58. 10.1016/S0140-6736(17)30938-8 28689664 PMC5592248

[6] European Centre for Disease Prevention and Control (ECDC). Workshop on Burden of RSV Disease in Europe. ECDC Expert Consultation Meeting, 23-24 November 2015, Stockholm. Stockholm: ECDC; 2015. Available from: https://www.ecdc.europa.eu/sites/portal/files/media/en/press/events/Documents/Meeting%20report%20ECDC%20RSV%20surv%20and%20burden%20of%20disease%20workshop%2023-24%20Nov.pdf

[7] The IMpact-RSV Study Group Palivizumab, a humanized respiratory syncytial virus monoclonal antibody, reduces hospitalization from respiratory syncytial virus infection in high-risk infants. The IMpact-RSV Study Group. Pediatrics. 1998;102(3 Pt 1):531-7. 9738173

[8] Direção-Geral da Saúde (DGS). Atualização de Norma DGS: Prescrição de Palivizumab para Prevenção de Infeção pelo Vírus Sincicial Respiratório em Crianças de Risco. [Update of Regulation DGS: Palivizumab Prescription for Prevention of Respiratory Syncitial Virus Infection]. Portugal: A Enfermagem e as Leis. [Accessed 21 Nov 2017]. Portuguese. Available from: http://www.aenfermagemeasleis.pt/2015/12/28/atualizacao-de-norma-dgs-prescricao-de-palivizumab-para-prevencao-de-infecao-pelo-virus-sincicial-respiratorio-em-criancas-de-risco/

[9] American Academy of Pediatrics Committee on Infectious DiseasesAmerican Academy of Pediatrics Bronchiolitis Guidelines Committee Updated guidance for palivizumab prophylaxis among infants and young children at increased risk of hospitalization for respiratory syncytial virus infection. Pediatrics. 2014;134(2):415-20. 10.1542/peds.2014-1665 25070315

[10] World Health Organization (WHO). Preferred Product Characteristics for Respiratory Syncytial Virus (RSV). Geneva: WHO; 2017. Available from: https://apps.who.int/iris/bitstream/handle/10665/258705/WHO-IVB-17.11-eng.pdf;jsessionid=9580BFC584AA121FFB822A82FECF1CA4?sequence=1

[11] Modjarrad K Giersing B Kaslow DC Smith PG Moorthy VS WHO RSV Vaccine Consultation Expert Group WHO consultation on Respiratory Syncytial Virus Vaccine Development Report from a World Health Organization Meeting held on 23-24 March 2015. Vaccine. 2016;34(2):190-7. 10.1016/j.vaccine.2015.05.093 26100926 PMC6858870

[12] Nyawanda BO Mott JA Njuguna HN Mayieka L Khagayi S Onkoba R Evaluation of case definitions to detect respiratory syncytial virus infection in hospitalized children below 5 years in Rural Western Kenya, 2009-2013. BMC Infect Dis. 2016;16(1):218. 10.1186/s12879-016-1532-0 27207342 PMC4875667

[13] Haynes AK Manangan AP Iwane MK Sturm-Ramirez K Homaira N Brooks WA Respiratory syncytial virus circulation in seven countries with Global Disease Detection Regional Centers. J Infect Dis. 2013;208(Suppl 3):S246-54. 10.1093/infdis/jit515 24265484

[14] Rha B Dahl RM Moyes J Binder AM Tempia S Walaza S Performance of Surveillance Case Definitions in Detecting Respiratory Syncytial Virus Infection Among Young Children Hospitalized With Severe Respiratory Illness—South Africa, 2009-2014. J Pediatric Infect Dis Soc. 2019;8(4):325-33. 10.1093/jpids/piy055 29931284 PMC12813577

[15] European Commission. Commission Implementing Decision (EU) 2018/945 of 22 June 2018 on the communicable diseases and related special health issues to be covered by epidemiological surveillance as well as relevant case definitions. 2018;(2119):1-74. Available from: https://eur-lex.europa.eu/legal-content/EN/TXT/PDF/?uri=CELEX:32018D0945&from=en

[16] Páscoa R Rodrigues AP Silva S Nunes B Martins C Comparison between influenza coded primary care consultations and national influenza incidence obtained by the General Practitioners Sentinel Network in Portugal from 2012 to 2017. PLoS One. 2018;13(2):e0192681. 10.1371/journal.pone.0192681 29438406 PMC5811043

[17] Gunson RN Collins TC Carman WF Real-time RT-PCR detection of 12 respiratory viral infections in four triplex reactions. J Clin Virol. 2005;33(4):341-4. 10.1016/j.jcv.2004.11.025 15927526 PMC7108440

[18] Mandrekar JN Receiver operating characteristic curve in diagnostic test assessment. J Thorac Oncol. 2010;5(9):1315-6. 10.1097/JTO.0b013e3181ec173d 20736804

[19] Broberg EK Waris M Johansen K Snacken R Penttinen P Influenza E European Influenza Surveillance Network Seasonality and geographical spread of respiratory syncytial virus epidemics in 15 European countries, 2010 to 2016. Euro Surveill. 2018;23(5). 10.2807/1560-7917.ES.2018.23.5.17-00284 29409569 PMC5801642

[20] Okiro EA Ngama M Bett A Nokes DJ The incidence and clinical burden of respiratory syncytial virus disease identified through hospital outpatient presentations in Kenyan children. PLoS One. 2012;7(12):e52520. 10.1371/journal.pone.0052520 23300695 PMC3530465

[21] Saha S Pandey BG Choudekar A Krishnan A Gerber SI Rai SK Evaluation of case definitions for estimation of respiratory syncytial virus associated hospitalizations among children in a rural community of northern India. J Glob Health. 2015;5(2):010419. 10.7189/jogh.05.020419 26649172 PMC4652925

[22] Durani Y Friedman MJ Attia MW Clinical predictors of respiratory syncytial virus infection in children. Pediatr Int. 2008;50(3):352-5. 10.1111/j.1442-200X.2008.02589.x 18533951

[23] Iwane MK Farnon EC Gerber SI Importance of global surveillance for respiratory syncytial virus. J Infect Dis. 2013;208(Suppl 3):S165-6. 10.1093/infdis/jit484 24265473

[24] Walsh EE Peterson DR Falsey AR Is clinical recognition of respiratory syncytial virus infection in hospitalized elderly and high-risk adults possible? J Infect Dis. 2007;195(7):1046-51. 10.1086/511986 17330796

[25] Volling C Hassan K Mazzulli T Green K Al-Den A Hunter P Respiratory syncytial virus infection-associated hospitalization in adults: a retrospective cohort study. BMC Infect Dis. 2014;14(1):665. 10.1186/s12879-014-0665-2 25494918 PMC4269936

[26] Nichol KL Mendelman P Influence of clinical case definitions with differing levels of sensitivity and specificity on estimates of the relative and absolute health benefits of influenza vaccination among healthy working adults and implications for economic analyses. Virus Res. 2004;103(1-2):3-8. 10.1016/j.virusres.2004.02.005 15163481

[27] Beyer WEP Heterogeneity of case definitions used in vaccine effectiveness studies--and its impact on meta-analysis. Vaccine. 2006;24(44-46):6602-4. 10.1016/j.vaccine.2006.05.038 16797799

